# Using drug scheduling to manage adverse events associated with hedgehog pathway inhibitors for basal cell carcinoma

**DOI:** 10.18632/oncotarget.28145

**Published:** 2021-12-21

**Authors:** John T. Lear, Reinhard Dummer, Alexander Guminski

**Affiliations:** ^1^Manchester Academic Health Science Centre, University of Manchester, Manchester, UK; ^2^Department of Dermatology, University Hospital, University of Zurich, Zurich, Switzerland; ^3^Skin Cancer Center, University Hospital, University of Zurich, Zurich, Switzerland; ^4^Department of Medical Oncology, Royal North Shore Hospital, St Leonards, Australia; ^5^Faculty of Medicine, Sydney Medical School, The University of Sydney, Sydney, Australia

**Keywords:** adverse events, basal cell carcinoma, sonidegib, hedgehog inhibitor, drug scheduling

## Abstract

Basal cell carcinoma (BCC) is the most common malignancy and form of skin cancer worldwide; advanced BCC, either as locally advanced BCC (laBCC) or metastatic BCC (mBCC), can cause substantial tissue invasion and morbidity. Until the recent availability of the hedgehog pathway inhibitors (HHIs) sonidegib and vismodegib, treatment options for advanced BCC were limited. These agents demonstrate efficacy in patients with laBCC and mBCC; however, the adverse events (AEs) associated with these agents can lead to treatment interruption or discontinuation and reduced quality of life, all of which significantly impact long-term adherence to therapy, which might affect clinical outcome. Given that most AEs are class-related effects, switching HHIs does not appear to lead to a significantly different AE profile, underscoring the importance of maintaining patients on their first HHI. Interrupting treatment of sonidegib and vismodegib does not appear to undermine the efficacy of these agents and is therefore a practical option to manage AEs in order to maintain continued treatment and disease control.

## INTRODUCTION

Basal cell carcinoma (BCC) is the most common malignancy and form of skin cancer worldwide [1, 2]. BCC accounts for most (~80%) of the approximately 3.5 million global annual diagnoses of nonmelanoma skin cancer [1]. BCC infrequently results in death or metastasis; however, advanced BCC, either as locally advanced BCC (laBCC) or metastatic BCC (mBCC), can cause substantial tissue invasion and morbidity [2, 3]. Severe (advanced) BCC accounted for <1% of all BCCs in almost 10,000 patients at 1 tertiary referral center [4]. Primary treatments for BCC include cryotherapy, curettage and electrodessication, surgical excision, Mohs micrographic surgery, and radiation therapy [5]. While these treatments are generally successful, treatment options for patients with advanced BCC are more limited. Moreover, since about 80% of BCCs appear on the head and neck [6], excision of locally advanced lesions in these areas can cause disfigurement and loss of function.

The hedgehog signaling pathway regulates normal cell development and proliferation and plays a key role in the development of BCC [1, 7–9]. In particular, most BCCs are associated with upregulation of the hedgehog signaling pathway due to mutations in the human homologue of the Drosophila patched (*PTCH1*) gene or smoothened (SMO) protein [1, 7, 9]. Inhibition of the hedgehog signaling pathway is therefore a rational treatment option in patients with advanced BCC. Vismodegib (Erivedge^®^; Genentech USA Inc.; South San Francisco, CA, USA) and sonidegib (Odomzo^®^; Sun Pharmaceutical Industries, Inc.; Cranbury, NJ, USA) are hedgehog pathway inhibitors (HHIs) that selectively target *SMO*. These drugs are approved by the Food and Drug Administration (FDA) and European Medicines Agency (EMA) for treatment of laBCC that has recurred following surgery or radiation therapy or those who are not candidates for surgery or radiation therapy; vismodegib has also been approved by the FDA and EMA for mBCC, and both agents are approved for treatment of mBCC in Australia and Switzerland [10–13].

While the HHIs are important treatment advances for patients with advanced BCC, a population with previously limited treatment options, the safety profile of these agents significantly impacts long-term adherence to therapy [14]. Adverse events (AEs) associated with HHIs can lead to treatment interruption or discontinuation and reduced quality of life, all of which might affect clinical outcome. Given that most AEs are class related, switching HHIs does not appear to lead to a significantly different AE profile [14]. This underscores the importance of maintaining patients on their first HHI. This article reviews the pivotal clinical trials in advanced BCC of vismodegib and sonidegib and provides clinical strategies for managing AEs to maximize benefit from therapy.

### Hedgehog pathway in BCC

The hedgehog signaling pathway is usually silenced in most normal adult tissues; however, it plays a pivotal role in repair of damaged tissues, promotion of stem cell proliferation, and regulation of maintenance of tissues including muscle, hair, taste buds, and the reproductive system [15–18]. The pathway can be stimulated by 3 hedgehog ligands, including Sonic hedgehog, Indian hedgehog, and Desert hedgehog; Sonic hedgehog is the most widely expressed in adult tissues and the most potent of these ligands [16, 19]. The primary receptor for these ligands is PTCH1 [9]. PTCH1 inhibits SMO in the absence of the hedgehog ligands, whereas ligand binding relieves the inhibitory effects of PTCH1 on SMO. SMO suppression is crucial for normal cell regulation and proliferation; activation of SMO signaling leads to activation of the glioma-associated oncogene (GLI) transcription factors GLI1, GLI2, and GLI3; GLI1 activates genes associated with tumorigenesis (eg, cyclin-D1*, MYC*, *BCL2*) and angiogenesis [16].

In BCC, the hedgehog signaling pathway is typically activated by mutations that lead to *PTCH1* gene dysfunction, which is almost universally observed in patients with BCC nevus syndrome and in most patients (80%–90%) with sporadic BCC tumors, whereas 10% of patients have *SMO* gene mutations [14, 18]. Both mutation types lead to constitutive SMO signaling and BCC development [18]. Activation of the Sonic hedgehog signaling pathway (by the deactivating mutation of PTCH or activating mutations of SMO) is therefore key to developing BCC.

### Hedgehog pathway inhibitors in BCC

The discovery of the importance of hedgehog pathway signaling in oncogenesis led to the development of the HHIs [20]. As noted above, there are currently 2 hedgehog inhibitors approved for use in advanced BCC: vismodegib and sonidegib. This section overviews the pharmacology and clinical efficacy and safety of these 2 orally available small-molecule drugs in advanced BCC (Table 1).

**Table 1:** Characteristics of vismodegib and sonidegib from pivotal trials used for approval [10, 11, 22, 32]

	Vismodegib (Erivedge)	Sonidegib (Odomzo)
**Pivotal trial for approval**	ERIVANCE phase 2 trial	BOLT phase 2 trial
**Date of approval**	FDA: January 2012 EMA: July 2013	FDA: July 2015 EMA: August 2015
**Dosage**	150 mg/day oral capsule	200 mg/day oral capsule
**Pharmacokinetics**	Oral bioavailability, 32% (single dose); 7% (multiple dose) Plasma protein binding, >97% Volume of distribution, 16.4–26.6 L Elimination half-life, 12 days (single dose); 4 days (multiple dose)	Oral bioavailability, <10% Plasma protein binding, >99% Volume of distribution, >9000 L Elimination half-life, 28 days
**Overall response rate**	39 months (investigator reported) [35] laBCC, 60.3% mBCC, 48.5%	42 months (central review) [37] laBCC, 56% mBCC, 8%
**Adverse events**	Muscle spasms, alopecia, dysgeusia, weight loss, fatigue, nausea, diarrhea, constipation, decreased appetite, arthralgias	Muscle spasms, alopecia, dysgeusia, fatigue, nausea, musculoskeletal pain, diarrhea, decreased weight, decreased appetite, myalgia

Abbreviations: EMA: European Medicines Agency; FDA: Food and Drug Administration.

### Pharmacodynamics

Ligand-independent pathway activation by SMO is one of the mechanisms believed to be involved in tumorigenesis [21]. Sonidegib and vismodegib are selective HHIs; they bind to and inhibit SMO, a transmembrane protein involved in hedgehog signal transduction [10, 22]. SMO inhibition results in transcription factors GLI1 and GLI2 remaining inactive, thus preventing expression of tumor-mediating genes within the hedgehog pathway [23].

In a phase I study in 33 patients with mBCC or laBCC, tumor *GLI1* was overexpressed as compared with normal skin controls; skin biopsies showed that *GLI1* expression was down-modulated more than 2-fold in 10 (77%) of 13 patients following treatment with vismodegib [24]. Vismodegib showed antitumor activity, with response seen in 18 (55%) of 33 patients; of the remaining 15 patients, 11 had stable disease and 4 had progressive disease. *GLI1* down-modulation did not correlate with vismodegib levels in individual patients.

*In vitro* studies and animal models demonstrate the targeted inhibition of hedgehog signaling and the antitumor activity of sonidegib; oral administration of sonidegib in mouse models resulted in complete suppression of *GLI1* expression and tumor regression [25, 26]. Sonidegib showed dose- and exposure-dependent inhibition of *GLI1* in BCC tumor and normal skin biopsies [27]. The observed tumor responses were associated with evidence of hedgehog pathway activation. In the phase 2 Basal Cell Carcinoma Outcomes with LDE225 (sonidegib) Treatment (BOLT) study in patients with laBCC who had valid biomarker samples, the median percentage of reduction in *GLI1* expression levels associated with sonidegib treatment (200 mg/day) was >90% at weeks 9 and 17 [28]. Moreover, the greatest reductions from baseline were observed in patients who had disease control, consistent with hedgehog pathway inhibition.

### Pharmacokinetics

Vismodegib has a mean absolute bioavailability of 32% following a single dose, and absorption was saturable, as evidenced by a considerably lower bioavailability (~7%) after continuous once-daily dosing [29]. The drug exhibits nonlinearity with respect to dose, as increasing the dosage from 150 mg once daily (the approved dose) to 270 mg or 540 mg once daily did not result in higher steady-state plasma concentrations; nonlinearity with respect to time was also observed, as steady-state plasma concentrations were achieved earlier than expected (typically within 7–14 days) and were lower than expected, based on single-dose pharmacokinetics [29–31].

Vismodegib is highly bound to plasma proteins (>99%), has a low volume of distribution (16–27 L), and is primarily eliminated by hepatic metabolism and biliary/intestinal excretion of unchanged drug (82% recovered in the feces and 4% recovered in urine) [22]. CYP2C9 appears to contribute in part to vismodegib metabolism [32]. The estimated terminal elimination half-life of vismodegib is about 12 days following a single dose and 4 days after continuous once-daily dosing [22], reflecting the increased clearance seen with repeated administration [33].

Sonidegib has a bioavailability of less than 10% following oral administration, but absorption increased 7 to 8 fold following administration with a high-fat meal [10]. The drug exhibits dose-proportional increases in maximum plasma concentrations over the dose range from 100 to 400 mg but less-than-proportional increases above 400 mg [11]. Steady-state drug concentrations are reached about 4 months after starting oral sonidegib [11]. The drug is highly bound (>97%) to plasma proteins and has a high volume of distribution (>9000 L); steady-state sonidegib levels are 6 fold higher in skin than in plasma [11].

Sonidegib is metabolized in the liver by CYP3A4; the main circulating compound is unchanged sonidegib (36%) and the circulating metabolites (45%) [11]. Sonidegib and its metabolites are mainly eliminated by the hepatic route, with about 70% eliminated in the feces and 30% eliminated in the urine [10]. The elimination half-life of sonidegib is about 28 days [11].

### Treatment response in BCC

ERIVANCE is an international, nonrandomized, single-arm, 2-cohort, multicenter trial that assessed the efficacy of vismodegib 150 mg daily in patients with laBCC and mBCC [34]. BOLT is an international, randomized, double-blind, multicenter trial of sonidegib 200 mg or 800 mg daily in patients with laBCC and mBCC [28]. ERIVANCE and BOLT both used objective response rate (ORR) by central review as the primary endpoint; ERIVANCE used conventional Response Evaluation Criteria in Solid Tumors (RECIST), while the more recent BOLT study used the more stringent modified RECIST (mRECIST) to assess BCC severity [28, 34]. Secondary efficacy endpoints included investigator-assessed best response, including complete response (CR), partial response (PR) and stable disease (SD), duration of response (DOR), progression-free survival (PFS), and overall survival (OS).

In ERIVANCE, 96 patients (63 with laBCC and 33 with mBCC) were evaluated for efficacy; 61% of patients were men, and median age was 62 years [34]. At the initial 9-month follow-up primary analysis (all patients had potential to be followed ≥9 months; the median duration of treatment was 10.2 months) [22]. ORRs in patients with laBCC were 42.9% (27/63) by independent review and 60.3% (38/63) by site investigator; ORRs for BCC mBCC were 30.3% (10/33) by independent review and 45.5% (15/33) by site investigator [34]. At the 39-month follow-up, ORRs (reported by investigator review only) were similar to those at the initial follow-up (60.3% (38/63) for laBCC and 48.5% (16/33) for mBCC); 20, 18, and 15 patients in the laBCC group had complete responses, partial responses, or stable disease, respectively, and the corresponding numbers in the mBCC group were 0, 16, and 14, respectively (all per investigator review) [35]. Median duration of response per investigator review was 26.2 months in the laBCC group (38 responders) and 14.8 months in the mBCC group (16 responders) [35].

Other major clinical studies with vismodegib include the SafeTy Events in VIsmodEgib (STEVIE) study, a phase 2, single-arm, open-label, multicenter study evaluating safety and efficacy of the drug (150 mg daily) in patients with advanced BCC (laBCC or mBCC) in a setting representative of clinical practice [6] and MIKIE, a phase 2, randomized, double-blind, regimen-controlled, multicenter study evaluating the safety and efficacy of intermittent vismodegib doses (150 mg daily for 12 or 24 weeks, placebo for 8 weeks, then 150 mg daily for 8 weeks) in patients with multiple BCCs [36]. In STEVIE, ORRs (investigator assessed) at week 73 were 68.5% in 1077 patients with laBCC and 36.9% in 84 patients with mBCC; CR rates were 33.4% and 4.8%, respectively [6]. In MIKIE, patients receiving the 12-week regimen (*n* = 116) achieved a 63% reduction in the number of BCCs and an 83% reduction in the size of BCCs; the corresponding values for the 24-week regimen (*n* = 113) were 54% and 69%, respectively [36].

BOLT enrolled 79 patients in the sonidegib 200 mg group (66 with laBCC and 13 with mBCC) and 151 patients in the 800 mg group (128 with laBCC and 23 with mBCC); median age was approximately 66 years, and 63% of patients were men [28]. In the initial primary efficacy analysis (median follow-up of 13.9 months; central review), 36.4% of patients (20/55) in the 200 mg group (42.9% (18/42) and 15.4% (2/13) in the laBCC and mBCC groups, respectively) and 33.6% (39/116) in the 800 mg group (37.6% (35/93) and 17.4% (4/23) in the laBCC and mBCC groups, respectively) achieved an objective response [28]. In the BOLT final analysis (42 months; central review), ORR was achieved in 56% of patients with laBCC and 8% of patients with mBCC in the 200 mg group and in 46% with laBCC and 17% with mBCC in the 800 mg group [37]. In the sonidegib 200 mg group, median duration of response for responders per central review was 26.1 months in the laBCC group and 23.3 months in the mBCC group; in the 800 mg group, median DOR was 24.0 months in the laBCC group and not estimable in the mBCC group [37].

### Adverse events

AEs associated with HHIs are very common and can be a significant limiting factor for continuous treatment. The median durations of exposure in ERIVANCE and STEVIE were 13 months [35] and 9 months [6], respectively. All patients experienced an AE in ERIVANCE; 58 (56%) experienced grade ≥3 AEs, 9 (9%) experienced serious treatment-related AEs, and 22 (21%) discontinued due to AEs [35]. The most commonly reported AEs were muscle spasms in 74 patients (71%), alopecia in 69 patients (66%), and dysgeusia in 58 patients (56%). In STEVIE, 1192 patients (98%) experienced an AE, 531 (45%) experienced grade ≥3 AEs, 289 (24%) experienced serious AEs, and 380 (31%) discontinued due to AEs [6]. Muscle spasms (*n* = 807; 66%), alopecia (*n* = 747; 62%), and dysgeusia (*n* = 663; 55%) were commonly reported AEs in STEVIE.

In MIKIE, median durations of vismodegib exposure were 71.4 weeks in the 12 week group and 68.4 weeks in the 24 week group; 33 patients (29%) and 40 patients (35%), respectively, experienced grade ≥3 AEs, and 6 (5%) and 2 (2%), respectively, experienced serious AEs related to treatment [36]. Discontinuations due to AEs were reported in 23 patients (20%) and 30 patients (27%), respectively, and the most common AEs (12- and 24-week groups, respectively) were muscle spasms (73% and 83%), dysgeusia (66% and 67%), and alopecia (63% and 65%).

The safety and tolerability profiles of sonidegib were more favorable for the approved 200-mg dose than those for the 800-mg dose. Median duration of exposure to sonidegib at the completion of BOLT was 11 months for the approved 200-mg dose and 6.6 months for the 800-mg dose [37]. Almost all patients (*n* = 77; 98%) in the 200-mg group experienced an AE. Grade ≥3 AEs were reported in 34 patients (43%) and 96 patients (64%) in the 200 mg and 800 mg groups, respectively; were treatment-related in 25 patients (32%) and 65 patients (43%), respectively; and led to discontinuation in 11 patients (14%) and 22 patients (15%), respectively [37]. The most commonly reported AEs in the 200 mg and 800 mg groups, respectively, were muscle spasms (54% and 69%), alopecia (49% and 58%), and dysgeusia (44% and 60%).

Overall, data from clinical trials show that vismodegib and sonidegib have similar tolerability profiles and that AEs such as muscle spasms, dysgeusia, and alopecia are class effects. Time to onset was typically within the first 6 months of vismodegib for these common AEs, with median times to onset of 1.9 months for muscle spasms, 3.4 months for alopecia, 1.5 months for dysgeusia, 2.8 months for fatigue, and 6.1 months for weight loss [38]. Median times to onset of common sonidegib AEs were 1.1 months for fatigue, 2.1 months for muscle spasms, and 6.5 months for weight loss [39].

In BOLT, the most common AEs for the 200 mg group at 30 months were muscle spasms (54%), alopecia (49%), and dysgeusia (44%); most AEs were mild, and the most frequent grade ≥3 AEs were elevated creatine kinase (CK) levels (6%), weight loss (5%), and muscle spasms (3%) [40]. It is worth noting that CK monitoring was not part of the ERIVANCE protocol and that the number of patients with CK elevations was not reported in the final analysis [35]. In STEVIE, 44 of 121 patients (36%) with >1 CK measurement who did not experience muscle spasms had elevated CK levels, and grade ≥3 elevations were reported in 4 patients (3%) [41]. Among 59 patients in STEVIE with >1 CK measurement who did experience muscle spasms, 20 (34%) had elevated CK levels and 2 (3%) had grade ≥3 elevations. In MIKIE, CK elevations were reported in 11 patients (10%) and 15 patients (14%) in the 12-week and 24-week regimen groups (grade 3 in 1% and 4% of patients, respectively) [36]. Therefore, muscle-related AEs and CK elevation also appear to be a class-related effect of HHIs.

In general, data from pivotal trials suggest that sonidegib has slightly less frequent and less severe AEs compared with vismodegib and that patients treated with sonidegib experience AEs slightly later than with vismodegib (with the exception of fatigue) [39]. Despite the majority of AEs reported in pivotal vismodegib and sonidegib trials being mild (grade 1 or 2 in severity), AEs were a primary reason for early discontinuation and frequently represent a limiting factor in continuous treatment [35]; therefore, dose adjustments or different schemes to avoid discontinuation and increase patient compliance are often required [42, 43]. Indeed, about 57% of patients in ERIVANCE discontinued vismodegib, 150 mg, by 39 months due to AEs or physician/patient decision [35], and the corresponding value for sonidegib, 200 mg, was about 53% at 30 months [39].

### Managing AEs associated with HHIs in BCC

The majority of AEs associated with HHIs typically appear after a few weeks from the beginning of treatment; therefore, patient education is vital for better management of these AEs and avoiding treatment discontinuation [44]. Due to the likelihood of AEs resulting in treatment discontinuation, treatment interruptions are frequently used as an approach to manage patient care, especially with AEs of greater severity [14]. Indeed, treatment delay/interruption or dose modification is an important aspect of hedgehog pathway inhibitor treatment for BCC in order for clinicians to best manage their patients’ therapeutic course. As noted above, AEs were the main cause of discontinuations from the pivotal vismodegib and sonidegib trials [35, 39].

For ERIVANCE, treatment with vismodegib could be interrupted for up to 4 weeks for evaluation of intolerable toxicity or for up to 8 weeks for a planned surgical procedure, and there were no planned dose reductions [34]. Patients with asymptomatic or tolerable severe AEs could continue to receive study drug if the AE was manageable and the patient and investigator agreed that continued treatment was acceptable. Exploratory post hoc analyses were conducted to evaluate the impact of missed vismodegib doses on ORR in ERIVANCE [35]. In the laBCC cohort, ORR was observed in 7 of 12 patients (58.3%) with no missed doses and in 31 of 49 patients (63.3%) who missed up to 33% of vismodegib doses (only 2 patients missed >33% of doses, both in the laBCC cohort); the corresponding ORRs in the mBCC cohort were 60.0% (6/10) and 43.5% (10/23), respectively [35]. In MIKIE, treatment interruptions for up to 2 weeks (up to 4 weeks total within the treatment phase) were permitted to manage toxic events; treatment interruption did not compromise the efficacy of vismodegib [36]. Vismodegib maintained efficacy in a post hoc analysis of data from ERIVANCE and STEVIE using a tumor growth inhibition model to evaluate the effect of 8-week treatment interruptions on efficacy in patients with laBCC and mBCC [45].

There are no comparable studies that evaluated the impact of dose interruptions of a prespecified length with sonidegib. The BOLT protocol recommended a number of dose modifications and dose delays for suspected treatment-related toxicities; these are summarized in Table 2. For example, in patients with normal CK and grade 1 to 2 muscle-related symptoms, patients continued sonidegib (ie, no dose reduction or delay). In patients with grade ≥2 muscle-related AEs, CK was measured weekly until symptoms resolved to grade ≤1, and for grade ≥3 muscle-related AEs, treatment was delayed. Additionally, in patients with asymptomatic or symptomatic grade 1 to 2 CK elevation, the same dose was continued (CK was monitored weekly until resolution to grade ≤1 in symptomatic CK elevation). However, in patients with grade 3 to 4 CK elevation, dosing was interrupted and laboratory and CK values were monitored weekly until resolution to grade ≤1; sonidegib treatment resumed at a reduced dose if renal function was not impaired and CK elevation resolved to grade ≤1 within 21 days.

**Table 2:** Recommended dose modifications and dose delays for suspected treatment-related adverse events^a^

Worst toxicity; CTCAE grade^b^	During a cycle of therapy
*General (eg, nausea, dysgeusia, decreased appetite, diarrhea, vomiting, etc)*	
Grade 1–2	Maintain dose level
Grade 3	Omit dose until resolved to grade ≤1, then decrease dose by 1 step
Grade 4	Omit dose and discontinue patient from treatment
*Muscle toxicity*	
Normal CK with muscle-related symptoms (eg, pain, spasms, cramps)	Grade 1–2 symptoms Continue sonidegib at same dose; consider symptomatic treatment for muscle-related toxicity Grade 3 symptoms Hold dose for ≤21 days; measure CK; resume sonidegib at a reduced dose if resolved or improvement to grade 1 occurs
Grade 1–2 CK elevation	Asymptomatic (no new onset or worsening of muscle cramps, myalgia, or other muscle symptoms) Continue sonidegib at same dose and draw blood for pharmacokinetic analysis Symptomatic Continue same dose; monitor CK at least weekly
Grade 3–4 CK elevation	Any occurrence Omit sonidegib dose Check blood and/or urine myoglobin Monitor renal function Measure CK at least twice weekly Consider electromyography and muscle biopsy Consider resuming sonidegib at a reduced dose if renal function is not impaired and resolution to grade ≤1 occurs in <21 days Discontinue patient for renal impairment (SCr >2 × ULN)
*Cardiac*	
Cardiac: prolonged QTc interval grade ≥3 (QTcF >500 ms or increase of >60 ms from baseline on ≥2 separate ECGs)	First occurrence Omit dose Perform a serum potassium and magnesium analysis; if below LLN, correct with supplements Perform a repeat ECG within 1 hour of the first QTcF of >500 ms If QTcF remains >500 ms, repeat ECG as clinically indicated but at least daily until QTcF returns to <480 ms Once QTcF prolongation has resolved, treatment may be restarted at a reduced dose level Second occurrence Discontinue patient from treatment
*General cardiac*	
Grade 1–2	Maintain dose level
Grade 3–4	Omit dose and discontinue treatment
*Hepatic: bilirubin*	
Grade 1 total bili >ULN <1.5 **×** ULN	Maintain dose level
Grade 2 total bili 1.5-3 **×** ULN	Omit dose until resolved to grade ≤1, then: If resolved in ≤7 days, maintain dose level If resolved in >7 days, decrease dose by 1 step
Grade 3 total bili >3.0-10.0 **×** ULN	Omit dose until resolved to grade ≤1, then decrease dose by 1 step
Grade 4 total bili >10.0 **×** ULN	Omit dose and discontinue treatment
*Hepatic: AST or ALT*	
Grade 1 (>ULN 2.5 × ULN)	Maintain dose level
Grade 2 (>2.5-5.0 × ULN)	Maintain dose level
Grade 3 (>5.0-20.0 × ULN)	Omit dose until resolved to grade ≤1, then: If resolved in ≤7 days, maintain dose level If resolved in >7 days, decrease dose by 1 step
Grade 4 (>20.0 × ULN)	Omit dose until resolved to grade ≤1, then decrease dose by 1 step
AST or ALT >3.0 × ULN and total bili >2.0 × ULN	Omit dose and discontinue treatment
*Renal*	
Grade 1 SCr >ULN <1.5 × ULN	Maintain dose level
Grade 2 SCr 1.5-3 × ULN	Omit dose until resolved to grade ≤1, then: If resolved in ≤7 days, then maintain dose level If resolved in >7 days, then decrease dose by 1 step
Grade 3 SCr >3.0-6.0 × ULN	Omit dose until resolved to grade ≤1, then decrease dose by 1 step
Grade 4 SCr >6.0 × ULN	Omit dose and discontinue treatment

Abbreviations: ALT: alanine aminotransferase; AST: aspartate aminotransferase; bili: bilirubin; CK: creatine kinase; CTCAE: Common Terminology Criteria for Adverse Events; ECG: electrocardiogram; LLN: lower limit of normal; SCr: serum creatinine; ULN: upper limit of normal. ^a^A maximum of 1 dose reduction (to placebo treatment) was allowed for patients receiving the 200-mg dose, after which the patient would be discontinued from study treatment if there was a need for further dose reduction. ^b^Common Terminology Criteria for Adverse Events (CTCAE Version 4.03) were used.

Dose modifications were permitted for patients who were unable to tolerate the protocol-specified dosing schedule or, in the event of toxicities suspected to be related to the study drug, to keep the patient on treatment. A maximum of 2 dose reductions were permitted in patients in the 800 mg group (400 mg and then 200 mg), and a maximum of 1 dose reduction (to placebo) was permitted in patients in the 200 mg group; if there was a need for further dose reductions, the patient was discontinued from study treatment. For patients with any dose interruptions or delays, in those who experienced the same toxicity following re-initiation of treatment, regardless of duration, the second re-initiation of study drug was resumed at a lower dose. Any dose interruption or delay of >21 days from the previous dose resulted in discontinuation from study treatment. It is important to note that for those receiving sonidegib, 200 mg (the approved dose for BCC), who required a dose reduction, the decreased dose was placebo treatment in BOLT. Dose reductions of sonidegib in clinical practice are therefore not a practical option for patients requiring dose adjustments due to treatment-related AEs. For this reason, treatment interruptions in these patients offer a viable and maintainable option to manage AEs while safeguarding continued course of treatment [46].

Overall, through 42 months of treatment with sonidegib, dose reductions were reported in 13 patients (17%) in the 200 mg group and in 55 patients (37%) in the 800 mg group; treatment delays were reported in 54 patients (68%) and 98 patients (65%), respectively [46]. Adverse events accounted for nearly all of the dose reductions and about 60% and 80% of the treatment delays in the 200 mg and 800 mg groups, respectively [46]. It is also important to consider the impact of dose reductions and delays on treatment response. A recent subgroup analysis of BOLT indicated that the ORRs and durations of response in patients with dose reductions or delays were similar to those for the overall study group. In the 200 mg group in BOLT, ORRs by central review (laBCC and mBCC combined) were 48.1% overall, 46.2% in patients with ≥1 dose reduction or delay, and 48.5% in patients without dose reductions or delays [46]. The corresponding ORRs in patients with laBCC were 56.1%, 50.0%, and 57.4%, respectively. Results for duration of response and PFS in the 200 mg group were also similar to those in the overall group and in the subgroups of patients with or without dose reductions or delays [46].

## CONCLUSIONS

The HHIs sonidegib and vismodegib are promising treatment options for patients with advanced BCC; however, AEs that occur during treatment with these agents can be difficult for patients to endure and can impact treatment adherence. Therefore, treatment with these agents needs to maintain a balance between disease control and potential adverse reactions. The common AEs observed in vismodegib and sonidegib clinical trials are class-related effects and include muscle spasm, alopecia, dysgeusia, nausea, decreased appetite, and fatigue. Muscle-related effects are also a class-related AE associated with HHIs, and therefore CK levels should be monitored. Additionally, monitoring electrolytes, renal function, and liver function tests is also an important part of treatment care for patients receiving HHIs. Treatment interruptions of vismodegib and sonidegib do not appear to negatively impact the efficacy of these agents and are a practical option for managing AEs in order to maintain a continued course of treatment.

## Abbreviations

AE: adverse event
BCC: basal cell carcinoma
CK: creatine kinase
CR: complete response
DOR: duration of response
EMA: European Medicines Agency
FDA: the Food and Drug Administration
GLI: glioma-associated oncogene
HHI: hedgehog pathway inhibitors
laBCC: locally advanced basal cell carcinoma
mBCC: metastatic basal cell carcinoma
mRECIST: modified Response Evaluation Criteria in Solid Tumors
ORR: objective response rate
OS: overall survival
PFS: progression-free survival
PR: partial response
RECIST: Response Evaluation Criteria in Solid Tumors
SD: stable disease
SMO: smoothened
STEVIE: SafeTy Events in VIsmodEgib
